# Characterization of patients with occupational allergy to two new epoxy hardener compounds

**DOI:** 10.1111/cod.14109

**Published:** 2022-04-19

**Authors:** Sari Suomela, Maria Pesonen, Katriina Ylinen, Kristiina Aalto‐Korte, Katri Suuronen

**Affiliations:** ^1^ Occupational Medicine Finnish Institute of Occupational Health (FIOH) Helsinki Finland; ^2^ Work Environment Laboratories Finnish Institute of Occupational Health (FIOH) Helsinki Finland

**Keywords:** 1,3‐benzenedimethanamine, *N*‐(2‐phenylethyl) derivatives: CAS no. 404362‐22‐7, 2‐methylpentane‐1,5‐diamine: CAS no. 15520‐10‐2, allergic contact dermatitis, hydrogenated formaldehyde benzenamine polymer: CAS no. 135108‐88‐2 or 152 857‐36‐8, m‐xylylenediamine: CAS no. 1477‐55‐0

## Abstract

**Background:**

The practical importance of two recently described epoxy hardener allergens—1,3‐benzenedimethanamine, *N*‐(2‐phenylethyl) derivatives (1,3‐BDMA‐D) and hydrogenated formaldehyde benzenamine polymer (FBAP)—as occupational allergens remains to be defined.

**Objectives:**

To describe patients diagnosed at the Finnish Institute of Occupational Health (FIOH) with positive reactions to 1,3‐BDMA‐D or FBAP.

**Methods:**

We searched FIOH's patch‐test files from January 2017 to December 2020 for patients examined due to suspected occupational contact allergy to epoxy compounds. We analyzed the patch‐test results and sources of exposure to various epoxy hardeners and focused on occupations, symptoms, and the sources of exposure to 1,3‐BDMA‐D and FBAP.

**Results:**

During the study period, 102 patients were examined at FIOH for suspected occupational contact allergy to epoxy compounds. Of these, 19 (19%) were diagnosed with contact allergy to 1,3‐BDMA‐D (*n* = 10) or FBAP (*n* = 12). The largest occupational group was sewage pipe reliners (*n* = 8). Seven different hardener products contained FBAP, whereas 1,3‐BDMA‐D was present in only one hardener used by spray painters.

**Conclusions:**

A substantial number of patients with suspected occupational epoxy resin system allergy tested positive to in‐house test substances of 1,3‐BDMA‐D and/or FBAP.

## Graphical abstract

We found 18% of the patients examined due to suspected occupational contact allergy to epoxy compounds to have allergy to new hardener compounds. Most, but not all, of our formaldehyde benzenamine polymer (FBAP)‐ or 1,3‐benzenedimethanamine, N‐(2‐phenylethyl) derivatives (1,3‐BDMA) –positive patients had a positive reaction to structurally related diaminodiphenylmethane (MDA) or m‐xylylenediamine (MXDA), respectively. If a patient exposed to epoxy products has a positive reaction to MDA with no explanation, it might be a cross reaction to FBAP.

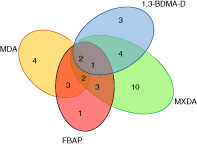

## 1 INTRODUCTION

Epoxy resin based on diglycidyl ether of bisphenol A (DGEBA‐R; CAS no. 1675‐54‐3 and CAS no. 25068‐38‐6) is the most frequent sensitizer in epoxy systems, but a considerable proportion of patients develop concomitant or solitary contact allergy to epoxy hardeners.^1^, ^2^, ^3^ Because allergic contact dermatitis (ACD) caused by epoxy products cannot always be diagnosed on the basis of patch testing with solely commercial test substances,^1^, ^4^ we patch test a selection of epoxy compounds as in‐house test substances. Furthermore, to identify new allergens in epoxy products, it is essential to test the patients' workplace products. This has led to novel contact allergens being identified in the epoxy hardener compounds 1,3‐benzenedimethanamine, *N*‐(2‐phenylethyl) derivatives (1,3‐BDMA‐D; CAS no. 404362‐22‐7),^5^ hydrogenated formaldehyde benzenamine polymer (FBAP; CAS no. 135108‐88‐2 or CAS no. 152857‐36‐8),^6^ and 2‐methylpentane‐1,5‐diamine (CAS no. 15520‐10‐2)^7^ in the past few years. To clarify the importance of these new epoxy hardeners as patch‐test substances and causes of occupational ACD, we studied patients who were being examined for suspected occupational ACD caused by epoxy products by patch testing with established epoxy system allergens and with FBAP, 1,3‐BDMA‐D, and 2‐methylpentane‐1,5‐diamine at our clinic in 2017 to 2020.

## 2 METHODS

At the Finnish Institute of Occupational Health (FIOH) clinic of occupational dermatology, all patients are examined for a suspected occupational skin disease. We perform patch testing using Finn Chambers (SmartPractice, Phoenix, AZ) in accordance with the ESCD guidelines.^8^ We read the tests two or three times (on day [D]2, D3, and D4; on D2, D3, and D6; or on D2 and D5), depending on the day of the week on which the patch tests were applied. We ask the patients to contact our clinic if they notice new reactions after the final reading. After patch testing, we examine occupational and nonoccupational (domestic) exposure to positive allergens in detail, on the basis of product information such as safety data sheets (SDSs) and labeling and, when necessary, by making inquiries to the manufacturers of the products.

For the present analysis, we searched the patch‐test and patient files from 2017 to 2020 for patients with positive reactions (+/++/+++) to the epoxy hardener compounds included in our epoxy patch test series and to the new epoxy hardener compounds 1,3‐BDMA‐D, FBAP, and 2‐methylpentane‐1,5‐diamine. These were included in an extension series that we tested on all patients who underwent the epoxy series testing. The epoxy test series contained the same test substances that we have described previously,^1^, ^4^ with only slight modifications. All the patients were tested with the baseline series, which includes DGEBA‐R. Most patients' patch tests also included their own products and materials from their workplaces.

**FIGURE 1:**
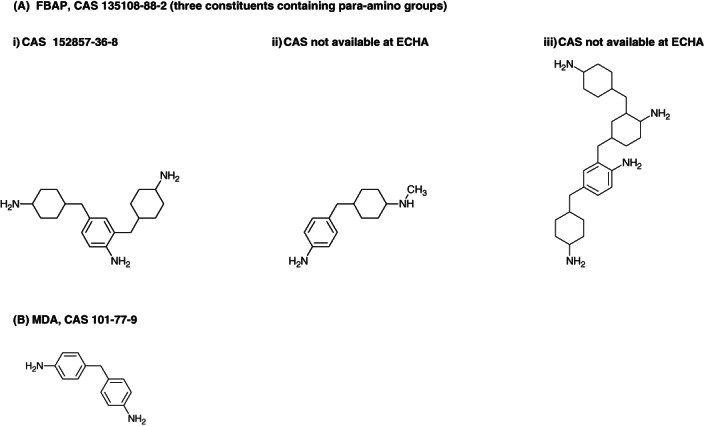
Molecule structures of (**A**) three recognized para‐amino structures contained in formaldehyde benzenamine polymer (FBAP) mixture and (**B**) diaminodiphenylmethane (MDA). FBAP contains also other substructures^14^

**FIGURE 2:**
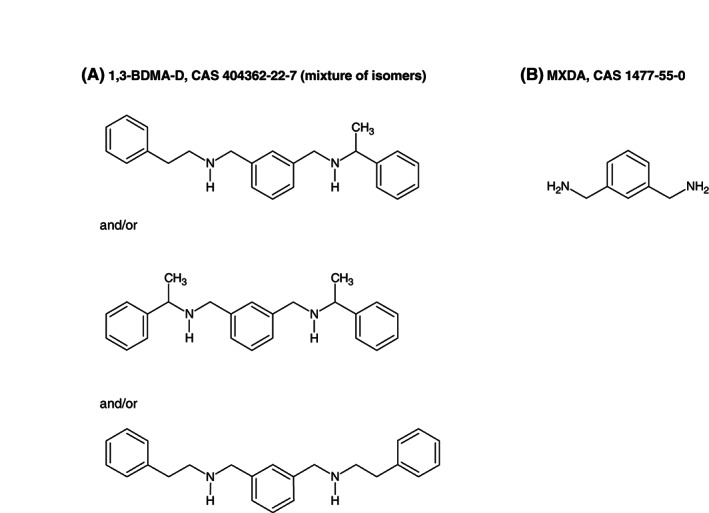
Molecule structures of (**A**) 1,3‐benzenedimethanamine, *N*‐(2‐phenylethyl) derivatives (1,3‐BDMA‐D), and (**B**) m‐xylylenediamine (MXDA)

Figures 1 and 2 show the chemical structures of the relevant hardeners. It is important to note that FBAP and 1,3‐BDMA‐D are mixtures, and that the molecular structures of their actual allergens are yet to be clarified.

## 3 RESULTS

During the study period, we patch tested a total of 102 patients with the epoxy series and its extension (Table 1). Nineteen (=19%) had positive reactions to FBAP (*n* = 12) and/or 1,3‐BDMA‐D (*n* = 10), whereas none of them reacted to 2‐methylpentane‐1,5‐diamine (Tables 1, 2). One irritant reaction was recorded for both FBAP and 1,3‐BDMA‐D. Twenty‐eight (27%) of the 102 patients tested positive to DGEBA‐R.

**TABLE 1:** Patch test reactions (+/++/+++) to selected hardener compounds and numbers of patients with confirmed exposure

Hardener compound	Test substance provider, conc. In pet.	Positive reactions *n* (% of total)	Confirmed exposure n/n of positive cases	Concomitant reaction to 1,3‐BDMA‐D n/n of positive cases	Concomitant reaction to FBAP n/n of positive cases	Concomitant reaction to DGEBA‐R/DGEBF‐R n/n of positive cases
FBAP CAS: no. 135108‐88‐2 or no. 152 857‐36‐8	In‐house, 1%	12 (12)	8/12	3/1	12/12	6/12
1,3‐BDMA‐D CAS: no. 404362‐22‐7	In‐house, 0.2%	10 (10)	2/10	10/10	3/10	5/10
2‐methylpentane‐1,5‐diamine CAS: no. 15520‐10‐2	In‐house, 0.25%	0	0	0	0	0
MXDA CAS: no. 1477‐55‐0	In‐house, 0.5%	22 (22)	20/22	7/22	8/22	12/22
IPDA CAS: no. 2855‐13‐2	AllergEAZE, 0.5%	5 (5)	5/5	2/5	2/5	2/5
Tris‐DMP CAS: no. 90‐72‐2	Chemotechnique, 0.5%	1 (1)	1/1	0	1/1	1/1

*Note*: N(total) 102 patients. Abbreviations: 1,3‐BDMA‐D, 1,3‐benzenedimethanamine, *N*‐(2‐phenylethyl) derivatives; DGEBA‐R, diglycidyl ether of bisphenol A resin, CAS no. 1675‐54‐3 and no. 25 068‐38‐6; DGEBF‐R, diglycidyl ether of bisphenol F resin, CAS no. 28064‐14‐4 and no. 9003‐36‐5; FBAP, hydrogenated formaldehyde benzenamine polymer; IPDA, isophoronediamine; MXDA, m‐xylylenediamine; pet., petrolatum; tris‐DMP, 2,4,6‐Tris‐(dimethylaminomethyl)phenol.

**TABLE 2:** Patients with allergic reactions to FBAP and 1,3‐BDMA‐D, to own hardener products containing FBAP or 1,3‐BDMA‐D, to other epoxy hardener compounds, and to MDA, MDI, or DGEBA‐R, and with dermatitis

Patient no.	Profession (gender/year of examination)	FBAP 1% pet.	1,3‐BDMA‐D 0.2% pet.	Patch test reactions to own hardener products	Presence of FBAP or 1,3‐BDMA‐D according to SDS	Reaction to other epoxy hardener compounds	Contact allergy to MDA, MDI, or DGEBA‐R	Location of dermatitis	Duration of work before symptoms (months)
1	Sewage pipe reliner (22y, M, 2017)	+†	Neg	BD/BDA 30: 1% (+) SprayCoat: 1% (neg)	BD/BDA 30: FBAP 10‐20% SprayCoat: FBAP‡	No	MDA^§^	Face, hand, wrist	3
2	Worker in wood processing industry (28y, F, 2019)	+++†	Neg	Colofil: 1% (+++); 0.32% (++)	Colofil: FBAP 10‐16%	No	DGEBA‐R† MDA^§^	Face, forearms, body, thighs	½
3	Sewage pipe reliner (50y, M, 2019)	+++†	++§	BD/BDA 30: 1% (+++); 0.1% (+++)	BD/BDA 30: FBAP 10‐20%	MXDA (++)†	DGFBA‐R† MDA^§^	Face, forearms, hands, decolté, thigh, leg	1
4	Sewage pipe reliner (20 y, M, 2017)	++†	Neg	SprayCoat: 1% (++) Amine mixture of SprayCoat: 1% (+++); 0.03% (+++)	SprayCoat: FBAP‡ Amine mixture of SprayCoat: FBAP‡	MXDA (++)†	DGEBA‐R† MDA^§^	Face, forearms, thighs	2–3
5	Sewage pipe reliner (21 y, M, 2018)	++†	Neg	SprayCoat: NT	SprayCoat: FBAP‡	MXDA (++)†	DGEBA‐R†	Face, forearms, hands, arm pits, thighs	6–9
6	Sewage pipe reliner (36 y, M, 2018)	++†	++§	BD/BDA 30: NT SprayCoat: NT BD/BDA 60: NT	BD/BDA 30: FBAP 10‐20% SprayCoat: FBAP‡ BD/BDA 60: FBAP 50‐75%	MXDA (+++)†	MDA MDI	Face, forearms, hands, wrists, body, thighs, legs	3
7	Floor layer (41 y, M, 2019)	++†	Neg	MasterTop P617B: 1% (++) Viasol EP T703B: 1% (+++); 0.32% (+)	MasterTop P617B: FBAP 40‐70% Viasol EP T703B: FBAP 20‐50%	MXDA (+++)† IPDA (+)†	No	Face, forearms	3
8	Floor layer (37 y, M, 2020)	+†	Neg	MasterTop P621B: NT	MasterTop P621B: FBAP 20‐75%	MXDA (++)†	No	Forearms, hands	12
9	Floor layer (32 y, M, 2018)	+§	Neg	NT with hardeners that contained FBAP	Not mentioned in SDSs	MXDA (+)†	MDA MDI†	Face, forearms, neck	1
10	Tile setter (37y, M, 2018)	+++§	Neg	NT with hardeners that contained FBAP	Not mentioned in SDSs	IPDA (+)†	DGEBA‐R† MDA MDI	Face, forearms, hands, knees, thighs, legs	½
11	Painter (34 y, M, 2020)	+	Neg	NT with hardeners that contained FBAP	Not mentioned in SDSs	Tris‐DMP (++)†	DGEBA‐R†	Face, forearms, hands, wrists, back, thighs, legs	1½
12	Spray painter (40 y, M, 2018)	Neg	+++†	Inerta 165‐01: 1% (+++); 0.1% (+++)	Inerta 165‐01: 1,3‐BDMA‐D 25‐50%	No	MDI	Face	18
13	Spray painter (32 y, M, 2017)	Neg	+++†	Inerta 165‐01: 1% (+++); 0.1% (++)	Inerta 165‐01: 1,3‐BDMA‐D 25‐50%	No	No	Face, hands	Not defined
14	Sewage pipe reliner (25 y, M, 2018)	Neg	++	Brawo I: 1% (++); 0.32% (+)	Brawo I: Not mentioned in SDSs, see text	MXDA (+)†	DGEBA‐R†	Face, forearms, wrist, neck	6
15	Sewage pipe reliner (21 y, M, 2018)	Neg	++	Brawo I: 1% (+++); 0.1% (+++) Brawo III: 1% (+++); 0.1% (++)	Brawo I and III: Not mentioned in SDSs, see text	No	DGEBA‐R†	Forearms, hands, thigh, knee	5
16	Floor layer (46 y, M, 2020)	IR	+++§	NT with hardeners that contained 1,3‐BDMA‐D	Not mentioned in SDSs	MXDA (+++)† IPDA (++)†	MDA	Ears, forearms, neck, wrists, knees	2
17	Sewage pipe reliner (23 y, M, 2017)	+	++§	NT with hardeners that contained FBAP or 1,3‐BDMA‐D	Not mentioned in SDSs	MXDA (++)†	No	Face, forearms, knees	5
18	Tile setter (28 y, M, 2018)	Neg	++	NT with hardeners that contained 1,3‐BDMA‐D	Not mentioned in SDSs	MXDA (+++) IPDA (+++)†	DGEBA‐R†	Face, forearms, neck, hands, wrists, thighs	1
19	Plywood worker (48y, M, 2017)	Neg	+	NT with hardeners that contained 1,3‐BDMA‐D	Not mentioned in SDSs	MXDA (++)	DGEBA‐R†	Face, forearms, hands, wrists	Not defined

*Note*: † = culprit allergen, § = possible cross reaction, ‡ = additional information from manufacturer.

Abbreviations: 1,3‐BDMA‐D, 1,3‐benzenedimethanamine, *N*‐(2‐phenylethyl) derivatives; DGEBA‐R, diglycidyl ether of bisphenol A ‐resin; FBAP, hydrogenated formaldehyde benzenamine polymer; IPDA, isophoronediamine; MDA, diaminodiphenylmethane; MDI, diphenylmethane‐4,4'‐diisocyanate; MXDA, m‐xylylenediamine; neg, negative; NT, not tested; SDS, safety data sheet; tris‐DMP, 2,4,6‐Tris‐(dimethylaminomethyl)phenol.

### 3.1 FBAP‐positive patients

Twelve patients had positive reactions to FBAP (Table 2). We have previously reported one of the six sewage pipe reliners (Patient 1).^6^ He was the only one of our FBAP‐positive patients who did not react to any epoxy compound other than FBAP and his own hardener product that contained FBAP.

Eight of the patients had used products that contained FBAP. Five of these were sewage pipe reliners. Seven different hardener products used by the FBAP‐positive patients contained FBAP according to the SDSs or additional information from the manufacturers (Table 2).

**FIGURE 3:**
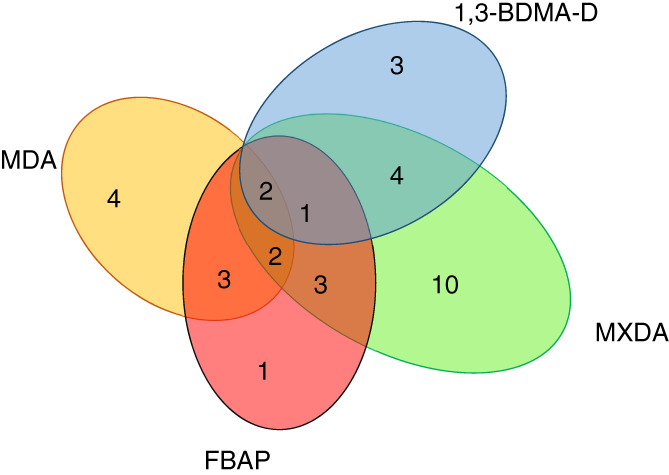
Venn diagram showing positive reactions to formaldehyde benzenamine polymer (FBAP), 1,3‐benzenedimethanamine, *N*‐(2‐phenylethyl) derivatives (1,3‐BDMA‐D), m‐xylylenediamine (MXDA), and diaminodiphenylmethane (MDA)

Six of the patients (50%) had a concomitant sensitization to DGEBA‐R. Seven FBAP‐positive patients (58%) had a concomitant positive reaction to diaminodiphenylmethane (MDA), whereas 64% (*n* = 7) of those allergic to MDA and tested with FBAP (*n* = 11) were positive to FBAP (Figure 3).

### 3.2 1,3‐BDMA‐D‐positive patients

Ten patients reacted to 1,3‐BDMA‐D (Table 2). Only two of them—two spray painters (Patients 12 and 13)—were clearly exposed to 1,3‐BDMA‐D. They also reacted to their own paint hardener that contained 1,3‐BDMA‐D. In addition, two sewage pipe reliners (Patients 14 and 15) with strong positive reactions to 1,3‐BDMA‐D had used hardener products that contained “unspecified amine adduct” with no announced CAS number in the SDSs. We did not manage to get this amine adduct for patch testing. There is a possibility that it might have resemblance to 1,3‐BDMA‐D.

Seven of the patients (70%) also reacted to m‐xylylenediamine (MXDA), and five of these had also used MXDA‐containing products. The other way round, 32% of those allergic to MXDA (*n* = 22) had a positive reaction to 1,3‐BDMA‐D (Tables 1, 2, Figure 3). Five of the patients (50%) had a concomitant positive reaction to DGEBA‐R (Tables 1, 2).

### 3.3 Other epoxy hardeners

Among the hardener chemicals we tested, MXDA elicited the largest number of allergic reactions (22%) (Table 1, Figure 3). Over 90% of the patients with allergy to MXDA had been exposed to products that contained MXDA. Five patients (5%) were allergic to isophoronediamine (IPDA) and had used IPDA‐ containing products. One painter was allergic to 2,4,6‐Tris‐(dimethylaminomethyl)phenol (tris‐DMP) and had used a hardener product that contained tris‐DMP (Tables 1, 2).

We detected sensitization to diethylenetriamine (CAS no. 111‐40‐0; 1% in pet.) and *N*‐aminoethylpiperazine (CAS no. 140‐31‐8, 0.25% in pet.) in two participants (no exposure found). Hardener compounds tetraethylenepentamine (CAS no. 112‐57‐2, 1.0% in pet.), triethylenetetramine (CAS: no. 112‐24‐3, 0.5% in pet.), dipropylenetriamine (CAS no. 56‐128‐8, 0.5% in pet.), and trimethylhexamethylenediamine (CAS no. 25620‐58‐0, 0.32% in pet.) were negative in all patients.

### 3.4 Symptoms and outcome of patients allergic to the two new epoxy hardeners

The hands/wrists/upper extremities were affected in all but one case (*n* = 18). Eleven patients had hand eczema, 16 had facial symptoms, and one patient had eczema on their ear lobes. Eleven patients (11/19) had eczema in their lower extremities.

The two spray painters with contact allergy to 1,3‐BDMA‐D were able to continue spray painting by working with other products, and another six patients were able to adapt their jobs. Ten patients (53%) with contact allergy to the new epoxy hardeners had to change occupations because of their occupational ACD. Of these, all but one had facial symptoms or eczema on their ear lobes, eight also had eczema in their lower extremities, and four had hand eczema. The one patient without facial symptoms had severe hand eczema and eczema on their knees and thighs.

## 4 DISCUSSION

Contact allergy to epoxy chemicals is the second most common cause of occupational ACD in Finland and has gained relative importance in the last decade.^9^ These chemicals include resins, reactive diluents, and hardener compounds, of which DGEBA‐R is the most common sensitizer.^1^ Here we describe a total of 19 patients sensitized to the new epoxy hardeners FBAP (12 patients) and 1,3‐BDMA‐D (10 patients). Our patients worked in sewage pipe relining, floor coating, industrial painting, and tile setting, all of which are occupations known to pose a high risk of skin exposure to epoxy chemicals.^1^, ^10^, ^11^, ^12^ In addition, two patients worked with either plywood or wooden boards and repaired the defects of the wood with epoxy putties.

Relevant exposure was detected in 8 of the 12 FBAP‐positive patients, revealing that several products had FBAP as an ingredient. According to the Finnish Chemical Product Register (FCPR), maintained by the Finnish Safety and Chemicals Agency, FBAP has been present in more than 200 epoxy products since 1997, and about 60 products that contain it are still on the market.^13^ The register might not even cover all the products on the market. Our preparation of 1% FBAP in petrolatum was able to detect many allergies with only one irritant reaction. Thus our test concentration seems to have been suitable.

FBAP is a complicated mixture with structural resemblance to MDA.^6^, ^14^ It consists of at least nine constituents of which at least three contain para‐amino structural parts (Figure 1). We have reported previously that allergic reactions to MDA are common among patients who use epoxy products, but specific exposure to MDA is difficult to trace.^15^ We also found that an allergic reaction to MDA most often represented contact allergy to diphenylmethane‐4,4'‐diisocyanate (MDI) or was a cross‐reaction to para‐amino compounds. Seven of our FBAP‐positive patients also had a positive reaction to MDA. We suspect cross allergy between FBAP and MDA. However, co‐sensitization might also be possible through co‐exposure to some unidentified compound. Unknown sensitization to FBAP is a possible explanation for the positive MDA reactions in the epoxy‐exposed patients in our previous study.^15^

In two spray painters, 1,3‐BDMA‐D was the cause of the occupational ACD. Four of our previously described six patients with occupational contact allergy to 1,3‐BDMA‐D were also spray painters and had been using this same hardener product. In addition, one production worker had handled ingredients of this hardener product in the manufacture of epoxy coatings.^5^ According to the FCPR, 1,3‐BDMA‐D is a rare ingredient in epoxy hardeners: only 4 products containing it are currently available, and about 10 products containing it have been on the market previously, in 2015 to 2021. In some patients, we were unable to confirm or rule out exposure to 1,3‐BDMA‐D, due to inaccurate information on the amine components of the hardener products in the SDSs.

1,3‐BDMA‐D structurally resembles an established allergen in epoxy resin systems, MXDA, otherwise known as 1,3‐benzenedimethanamine (1,3‐BDMA) (Figure 2). We suspect cross allergy between 1,3‐BDMA‐D and MXDA due to the high number of co‐reactions. However, the possibility of co‐exposure to both hardener compounds cannot be ruled out. Our test concentration of 1,3‐BDMA‐D was 0.2% in petrolatum, which elicited only one irritant reaction.

We detected no sensitization to the hardener compound 2‐methylpentane‐1,5‐diamine in our patient material. A German study found 2‐methylpentane‐1,5‐diamine in as few as 48 of 1850 products, which suggests that it is not a widely used hardener compound in epoxy resin systems.^7^ According to the FCPR, 2‐methylpentane‐1,5‐diamine is present in about 20 epoxy hardeners currently on the Finnish market.

As regards epoxy hardeners, MXDA elicited the greatest number of positive reactions, which is in line with the results of previous studies.^1^, ^4^, ^16^ Most of our patients with allergies to either of the new epoxy hardener compounds had a concomitant reaction to MXDA and had been using products containing MXDA.

In line with our previous studies,^1^, ^5^, ^6^ eczema on the face was common among patients who were sensitized to epoxy hardeners, as it was present in nearly all of the patients with contact allergy to FBAP or 1,3‐BDMA‐D. Facial symptoms may represent airborne contact dermatitis, which is common among patients who are allergic to various epoxy compounds.^17^, ^18^ Skin contact with epoxy resin system components may occur through direct contact with products but also via contaminated workplace surfaces, personal protective equipment, and recently hardened epoxy materials.^12^ Most of the patients developed eczema within a short time—only a few weeks or months after starting a job involving exposure to epoxy products. Not only the hands, upper extremities, and face were commonly affected: over half of the patients even had eczema on their lower extremities. This suggests that skin protection fails very easily, and that extreme care should be taken to avoid contamination of the skin and workplace surfaces with epoxy chemicals. It is notable that over half of the patients had to change their trade because of severe work‐related skin symptoms reoccurring on the face in particular.

In conclusion, 1,3‐BDMA‐D and FBAP were relevant contact allergens in this series of Finnish patients with occupational ACD caused by epoxy products. Mono‐allergies to these hardeners occurred in solitary patients, but FBAP was found to be an additional cause of ACD in a larger number of cases. Testing of these new substances requires in‐house test substances. If a patient exposed to epoxy products has an allergic reaction to MDA with no explanation, it is important to consider that this might be a cross‐reaction to FBAP. In such cases, FBAP should be searched in the products.

## CONFLICT OF INTEREST

No exterior funding. No conflict of interest to be declared.

## AUTHOR CONTRIBUTIONS

**Sari Suomela:** Conceptualization (equal); data curation (lead); writing – original draft (lead). **Maria Pesonen:** Conceptualization (equal); data curation (equal); writing – review and editing (equal). **Katriina Ylinen:** Data curation (equal); writing – review and editing (supporting). **Kristiina Aalto‐Korte:** Conceptualization (equal); data curation (equal); writing – review and editing (lead). **Katri Suuronen:** Conceptualization (equal); data curation (equal); writing – original draft (supporting); writing – review and editing (equal).

## Data Availability

The data that support the findings of this study are available on request from the corresponding author. The data are not publicly available due to privacy or ethical restrictions.
